# A study to investigate the effectiveness of SimMan® as an adjunct in teaching preclinical skills to medical students

**DOI:** 10.1186/1472-6920-14-231

**Published:** 2014-11-19

**Authors:** Meenakshi Swamy, Marina Sawdon, Andrew Chaytor, David Cox, Judith Barbaro-Brown, John McLachlan

**Affiliations:** School of Medicine, Pharmacy and Health, Holliday Building, Durham University, Queen’s Campus, University Boulevard, Stockton on Tees, TS17 6BH UK

**Keywords:** Clinical skills, SimMan®, Preclinical medical students

## Abstract

**Background:**

Following the GMC’s report on Tomorrow’s Doctors, greater emphasis has been placed on training in clinical skills, and the integration of clinical and basic sciences within the curriculum to promote the development of effective doctors. The use of simulation in the learning environment has the potential to support the development of clinical skills in preclinical medical students whilst in a ‘safe’ environment, but currently there is little evidence on its effectiveness.

**Methods:**

Seventy nine year one medical students were divided into two groups. A pre-test was conducted by both groups, after which one group performed chest examination on their peers whilst the other group examined the SimMan® manikin. Both groups subsequently performed a mid-test and crossed over so that the group that conducted peer examination examined the manikin and vice-versa. Finally a post-test was conducted. The students were scored for formative feedback whilst performing examinations. Students completed a feedback questionnaire at the end of the session. Data were analysed using a one-way ANOVA, independent *t*-test and 2- proportion Z test.

**Results:**

When the two groups were compared, there was no significant difference in their pre-test and post-test knowledge scores, whereas mid-test knowledge scores increased significantly (P < 0.001), with the group using SimMan® initially scoring higher. A significant increase in the test scores was seen in both groups after using SimMan® (P < 0.001). Students’ confidence increased significantly in differentiating between normal and abnormal signs (P < 0.001). Students highly valued the use of the manikin in the session with 96% of students reporting that it enhanced their learning experience.

**Conclusions:**

The study demonstrated a significant improvement in the students’ knowledge after examining the manikin and students also reported an increase in their confidence. Students’ feedback was generally very positive and they perceived the incorporation of manikin-based examinations useful to prepare them for future patient contact. The use of simulation in this context supports an integrated learning approach when used as an adjunct to peer examination, and can benefit the acquisition of clinical skills in preclinical medical students.

## Background

Over recent years, the early introduction of clinical skills teaching, and the integration of patient-focused clinical and basic sciences, has gained greater importance in undergraduate medical education. At Durham University, first and second year medical students practice clinical skills on each other under direct supervision from tutors, and receive immediate feedback on their performance during the clinical skills training sessions. They are introduced to a range of generic clinical skills, such as communication, clinical examination, and practical procedures, all of which are assessed during the summative Objective Structured Clinical Examinations (OSCEs). Students are trained so that they can subsequently perform these skills competently and confidently on patients. However, the challenges faced by preclinical students (Phase 1 medical students) are that they must learn and develop these clinical skills with limited exposure to patients, and also be able to apply these skills appropriately when they move on to clinical rotations. Several studies have documented that this transition from preclinical to clinical phases is stressful for medical students [1–4]. One of the areas in which both students and instructors feel there are difficulties is in the competent performance of clinical skills at an early stage in their clinical rotations [1].

Simulation-based education is seen to supplement the clinical education of medical students in a safe and supportive manner. It is a rapidly developing area which provides the learner with opportunities to repetitively practice, develop and transfer clinical skills without any fear of harm to patients, in a controlled setting. It is being used to augment learners’ knowledge and help them shape the acquisition of clinical skills [5–8]. Although simulation does not exactly duplicate clinical challenges or experiences with real patients in a genuine clinical setting, it complements students’ learning, and literature suggests that it can be used to prepare students for contact with real patients [5].

Laerdal SimMan® [9], a moderate fidelity manikin, is currently used widely in the later years of medical education. Students have evaluated its use positively in a number of studies, such as teaching undergraduate medical students in the context of management of medical emergencies [10–12], and the development of preclinical skills [13]. It provides learners with the opportunity to experience a learning environment as close as possible to clinical practice by providing physical representations of patients. It gives them the benefit of exposure to pathological states with the holistic experience of examining and treating a patient, without exposing them to real patients. Thus it can be used to bridge the gap between preclinical and clinical settings.

The aim of the study was to explore the role of SimMan® as an adjunct to peer examination in the teaching of clinical skills to preclinical medical students.

## Methods

Ethical approval was obtained from the School of Medicine, Pharmacy and Health, Durham University (ESC2/2013/PP003).

One hundred and four first year medical students at Durham University were invited to participate, of which seventy nine students volunteered and were included in the study. All students were taught the clinical skills of respiratory and cardiovascular system examination, and had previously performed these examinations on their peers.

Preclinical medical students at Durham University practice clinical skills on each other in a safe and supportive environment, the clinical skills laboratory. Students performed chest examination on each other and examined the manikin in a similar clinical setting in order to create a more realistic environment. The manikin can be programmed with a range of clinical examination findings [9, 13]. In this study, SimMan® 3G was programmed to display abnormal signs in each of the selected respiratory and cardiovascular clinical conditions based on the under-graduate medicine case-led curriculum (Table 1). Two SimMan® 3G manikins were set up in the clinical skills laboratory and were connected to equipment such as monitors and a simulated oxygen supply.

**Figure 1:**
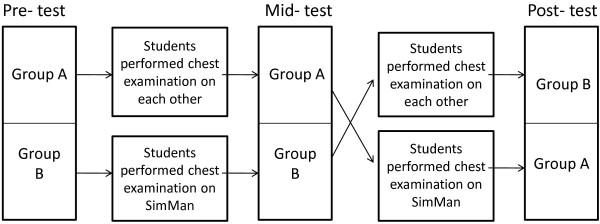
**A flow chart demonstrating the cross over study design.**

The students were randomly divided into two groups (A and B). Within each group they were further divided into subgroups consisting of two students who were facilitated by a tutor. The subgroups were created based on students’ preference choice of their peers with whom they were comfortable in performing examination. A pre-test was conducted at the beginning of the session with a questionnaire consisting of seven knowledge-based questions (with the maximum score of twelve), and three confidence level questions rated using a 4-point Likert scale (1-not at all confident to 4- very confident). In pairs, students performed chest examination and received immediate feedback from a tutor, the session lasting for approximately 20 minutes. Group A students used peer examination initially whilst Group B students initially examined the manikin. The students then answered the same questionnaire used in the pre-test as a mid-test, followed immediately by Group A examining the manikin and Group B examining each other. Finally a post-test (once again, with the same questionnaire) was conducted. The tutors scored students’ performance formatively using an objective scoring scheme (with the maximum score of twenty) similar to that used to assess them during formal summative clinical examinations. The students completed a feedback questionnaire at the end of the session. The cross over study model was used (Figure 1) in order to provide equivalent learning opportunities for all students. Thus all students performed examination both on their peers as well as on the manikin.

**Table 1:** **Clinical conditions with corresponding signs used in the study**

Clinical conditions	Signs
Chronic obstructive pulmonary disease	Increased respiratory rate, cyanosis and wheezy coarse crackles
Pulmonary regurgitation with atrial fibrillation	Murmur, irregular pulse and increased heart rate

Anonymized data was analyzed with specific statistical analyses, described within the results for each stage of the study. Quantitative data was analyzed using a one-way ANOVA and an Independent *t*-test. Qualitative data from confidence questions were analyzed using a 2-proportion Z test and Fischer’s exact test. A P-value < 0.05 was considered statistically significant in all tests. Qualitative data from the free text comments on feedback questionnaires was analyzed using thematic analysis. From the free text comments, the relevant data was coded into themes for each of the questions. Two authors (MSw and MS) independently coded the data and any differences were then discussed to reach an agreement.

## Results

### Knowledge

When the two groups were compared, it was found that the knowledge test scores (Table 2) did not differ significantly between Group A (using peer examination initially) and Group B (using SimMan® initially) for the pre-test (Independent *t*-test, t = 0.602, P = 0.549). Thus the baseline level of knowledge of both groups was considered to be equal. In the mid-test, Group B scored significantly higher after performing the examination on the manikin when compared to Group A who had performed peer-examination (Independent *t*-test, t = 5.411, P < 0.001). The knowledge test scores did not differ significantly between the two groups for the post-test. This suggests that their knowledge had improved equally after both groups had performed examination on the manikin (independent *t*-test, t = 0.109, P = 0.913).

**Table 2:** **Knowledge test scores from Group A (Group using peer examination initially) and Group B (Group using SimMan® initially)**

Tests	Groups	Mean	Standard deviation
Pre-test	A	6.7	1.43
	B	6.5	1.91
Mid-test	A	7.2	1.41
	B	9.3*	2.00
Post-test	A	9.4^†^	1.39
	B	9.3	1.96

Note: *denotes p < 0.001 between Group A and B for the mid-test.

^†^denotes p < 0.001 between Group A mid-test and Group A post-test.

Statistical analysis (one-way ANOVA) indicated that there was a significant difference between the pre-test and mid-test scores of Group B (F = 27.120, P < 0.001) and between mid-test and post-test of Group A (F = 39.857, P < 0.001). Further analysis (Independent *t*-test) confirmed that there was a significant increase in the mid-test scores (after performing examination on the manikin) when compared with the pre-test scores in the Group B (Independent *t*-test, t = 6.354, P < 0.001). In addition, post-test scores (after performing examination on the manikin) of Group A were significantly higher when compared with mid-test scores (Independent *t*-test, t = 6.928, P < 0.001) in the Group A. Thus students scored higher in the knowledge test after examining the manikin when compared to students who performed the examination on each other.

### Performance

Students scored higher when they performed examination on their peers rather than the manikin (Table 3). When student’s performance scores on each other were compared before and after simulation, it was found that the students scored higher after examining the manikin. The finding that Group B scored better on peer examination could be explained by the fact that they had more training (SimMan® and Peer examination) than Group A which had been exposed only to peer examination. After having previously performed examination (in previous teaching sessions, see methods) on their peers, students scored significantly higher by examining the manikin initially and then their peers (Independent *t*-test, t = 3.895, P < 0.001) as opposed to examining their peers initially, and then the manikin. Thus the manikin, when used as an adjunct to peer examination, can help students’ improve their ability to perform chest examination.

**Table 3:** **Performance scores during chest examination**

	SimMan® (Mean ± SD)	Peer examination (Mean ± SD)	
**Group A**	14.85 ± 2.94	15.87 ± 4.39	P = 0.22
**Group B**	13.33 ± 4.01	16.43 ± 2.93	P < 0.001

Group A (Group using peer examination initially); Group B (Group using SimMan® initially).

### Confidence

The mean confidence increased from the pre-test to mid-test, and then further increased in the post-test for both groups. The number of students who were confident in examining a patient increased significantly after examining their peers when compared to those after performing examination on the manikin, for both groups [Group A, pre-test (15) vs. mid-test (27), P = 0.007 and Group B, mid-test (18) vs. post-test (31), P <0.001)]. Students in both groups felt that they were more confident in differentiating between normal and abnormal signs after performing examination on the manikin [Group A, mid-test (13) vs post- test (28), P = 0.02 and Group B, pre-test (3) vs. mid-test (19), P <0.001)], but were more confident at performing examination in a clinical setting after peer examination [Group A, pre-test (4) vs. mid-test (24), P < 0.001 and Group B, mid-test (24) vs. post-test (33), P = 0.01)].

### Evaluation

Seventy eight (99%) students completed the feedback questionnaire (Table 4). Their responses to open-ended questions were analysed as follows:

**Table 4:** **Evaluation results of students’ feedback questionnaire**

	1	2	3	4	5
	Strongly disagree	Disagree	Unsure	Agree	Strongly agree
1. I feel the use of SimMan® to practice examination was useful	1%	3%	9%	60%	27%
2. SimMan® improved my knowledge for performing examination	1%	8%	20%	48%	23%
3. SimMan® reinforced the importance of being able to recognize normal findings	4%	4%	14%	51%	27%
4. I received useful feedback on my performance	0%	0%	1%	32%	67%
5. The session which included both SimMan® and peer examination enhanced my learning experience	0%	0%	4%	33%	63%
6. The session which included both SimMan® and peer examination made me feel more prepared to examine real patients	0%	4%	9%	59%	28%
7. The session was enjoyable	0%	2%	5%	49%	44%

1. I feel the use of SimMan® to practice examination was useful. Please state why?

The following themes emerged from the data:-

- Students who responded positively:
- Abnormal signs and external equipment useful

Most students valued the opportunity to examine abnormal signs and interpret the different parameters displayed on the monitor, which was not provided by the peer examination. They felt that it helped for better recognition of normal findings.

*“It was really helpful to hear/feel what was abnormal which I wouldn’t necessarily be able to do with my fellow students comparison”*

*“Allows you to examine abnormalities at this early stage. Being able to hear abnormal findings allows you to better recognise normal”*

*“Was good practise, useful as well seeing the monitors and dealing with clinical signs”*

*“It is useful to examine in a more clinical context, with issues occurring around SimMan and to listen to abnormal sounds”*

(ii) Improved clinical contextualisation

Students indicated that the manikin added clinical contextualisation to their learning. It provided them with an opportunity to perform examination in a simulated clinical setting.

*“It was realistic and placed me in a clinical like scenario. It allows me to challenge myself with a new setting”*

*“It is useful to examine in a more clinical context, with issues occurring around SimMan and to listen to abnormal sounds”*

*“He is able to simulate real life abnormal patients which is very useful for our hospital practice.”*

(iii) Increased perceived level of confidence

Students felt more confident in examining a patient after examining SimMan®.

*“I couldn’t remember any examination procedure but after the SimMan session I am more confident when carrying out the real patient examination”*

*“Improved my confidence in examining a patient. It showed me what to expect when looking and listening for abnormalities”*

(iv) Absence of realism

Students saw the absence of realism as a benefit when compared to examining their peers/patient.

*“It’s not a real patient and so if you get it wrong there’s no shame”*

*“Freedom to palpate”*

*“Also not the shyness/inhibitions associated with friends”*

*“Being able to examine a patient that isn’t a peer. It makes the experience more*

*serious”*

*“Very clear clinical signs and easier to examine than a real patient because I was less nervous”*

However, some thought that it was a drawback due to lack of interaction, immobility and inability to perform some parts of examination.

b) Students with negative/neutral response:
(i) **Absence of realism**

*“Otherwise the lack of interaction with the patient makes it difficult. Also SimMan is immobile which makes examination awkward”*

“*but it was difficult to practice communication skills”*

The negative/neutral response of students was mainly because they felt that there was absence of realism which impacted negatively on their experience of encountering the manikin although some of them found the monitor and abnormal signs useful.

*“It was intimidating and could not perform a full examination e.g. cap refill, clubbing which threw me and I panicked. There is no interaction with the ‘patient’.”*

*“The stats monitor was good but otherwise can’t recreate human experience.”*

*“It was difficult to imagine he was a real patient. And doing the normal tests – checking for clubbing was difficult as unrealistic”.*

(ii) Lack of exposure

One student commented that it was an “*amazing hardware*” but lack of exposure to the manikin did not render it very useful and felt frequent use would be better.

- SimMan® improved my knowledge for performing examination. Please state why?
- Students with positive response:
- Learnt about abnormal signs

Most students felt that the manikin provided them with an opportunity to learn about abnormal signs by examining it. It informed them how abnormal signs sound and feel. Students felt it enabled them to better differentiate between normal and abnormal.

*“It improved my knowledge of what heart murmurs and wheezing sounded like”*

*“He allowed us to hear what abnormal heart and lung sounds sounded like”*

*“I feel I’d be better at identifying abnormal”*

*“There’s some clinical knowledge we haven’t been taught but I picked them up during the SimMan® session.”*

(ii) Interpret monitor parameters

Students felt that they learnt to interpret different parameters displayed on the monitor and realised the importance of linking information.

*“I actually got to clearly relate my findings to the monitor’s data e.g. I linked heart value auscultation with an ECG”.*

*“Also importance of taking pulse even though got it on a screen.”*

(iii) Useful Feedback

Students felt that the feedback they received after examining the manikin was valuable.

*“Getting feedback on how to examine a non-healthy person – sometimes you can’t do the same things”*

(iv) Prepared students for future patient contact

Some students felt that using the manikin helped in the application of knowledge and increased their awareness of the pros and cons of examining a patient in clinical practice.

b) Students with negative/neutral response:
(i) Other methods were as good or better

*“ It showed me what to expect when looking and listening for abnormalities”*

*“Enabled me to understand the difficulties of performing CVR and respiratory examinations on more critically ill patients”*

*“The setting was like in a hospital. The SimMan has oxygen mask attached and he can be programmed to have simulated clinical problems. This allows me to understand the difference between normal and abnormal”*

Students felt they did not learn anything more than what they already knew. Some of them thought peer examination/examining patients provided more information than when using the manikin. However, some students felt the use of the manikin informed them about abnormal signs and external equipment.

“*For abnormal knowledge it was good but the rest could be taught in either setting”*

*“Not the same as a real person but useful to hear/feel abnormalities”*

(ii) Lack of exposure

One student mentioned lack of exposure was not very beneficial.

*“Interesting, but I feel it would be more useful with continued exposure since a one-time exposure to clinical settings is intimidating more than it is useful”*

5. The session which included both SimMan® and peer examination enhanced my learning experience. Please state why?

The majority of the students agreed or strongly agreed that the session which included both the manikin and peer examination enhanced their learning experience. The remaining students were unsure.

Students’ comments were overwhelmingly positive.

*“Good mix”*

*“Really good”*

*“Best of both worlds”*

Students found the experience of examining in different settings useful as there was deliberate practice and feedback on performance, it encouraged reflection and identified areas of improvement whist providing an opportunity to examine and identify normal and abnormal signs. Some students commented that it helped them remember the examination procedure/steps better, improved the appreciation of the importance of clinical examinations, increased confidence, encouraged checking for abnormality, and provided useful revision.

They considered peer examination better for performing examination and interaction whereas the manikin was preferred for the examination of abnormal findings and to help avoid uncomfortable mistakes. One student commented,

**“*Either alone has limitations but together they cover everything*”**

Students enjoyed examining peers and examining the manikin. Most students specifically mentioned they enjoyed examining abnormal signs and also getting constructive, useful and informative feedback. They felt this improved their knowledge and confidence. Specific comments were made regarding the manikin.

*“SimMan as it was really realistic”*

*“Use of SimMan to see abnormalities as it simulates reality in a pretty accurate manner”*

*“More exciting to investigate abnormalities, however this may be due to novelty”*

However, some students disliked the absence of realism in SimMan®.

*“Difficult to show empathy”*

It was interesting to note that one student commented, “*Cannot replace real patient contact but it’s the next best thing”* and another student felt “*SimMan’s clinical setting portrayed was more accurate but is intimidating rather than useful unless it is repeated”.*

The session stimulated students’ interest in recognising abnormalities, working with simulated patients, revision and clinical skills/practice.

Generally, students considered the session was very useful. Some of the general comments were:

*“Very enjoyable. Recommend for next year”*

*“Using peer examination and SimMan® is the best way forward, provides different things”*

*“Integrate into OSCE”*

## Discussion

In this study we have compared two teaching modalities - peer examination and examination using SimMan®. It was found that students’ knowledge required to perform respiratory and cardiovascular examination improved significantly after they performed examination on the manikin. However, it is interesting to note that repeated exposure to peer examination did not improve students’ knowledge. In addition, the manikin was as effective as peer examination in increasing students’ confidence. These findings are similar to other studies that have reported an improvement in preclinical medical students’ knowledge and confidence after simulation [12, 13]. We found a significant increase in students’ knowledge after examining the manikin in both groups whereas in the study conducted by Swamy et al. [13], there was a similar, non-significant trend in the group initially using peer examination. One of the advantages of simulation is that it allows application of theoretical knowledge to practice [11]. When students perform examination on each other, they become familiar with normal examination findings but they are aware that they are examining a healthy person. SimMan®, on the other hand provides students with the variability of examining both normal and abnormal clinical signs, which reflects more closely what they are likely to encounter in clinical practice. During examination this encourages students to actually look for signs rather than just comment that they were either present or absent. The manikin provides them with the opportunity to apply their knowledge and thus reinforces normal findings; focusing them towards looking for abnormalities or deviations from normal. One student aptly summarised this, saying *“I was able to explore the abnormal and know the abnormality. In peer examination normal is always ‘assumed’ rather than checked. SimMan® you don’t know for sure that you are examining a normal patient”*. Hence most students found the manikin useful for practicing examination and felt that it added clinical contextualisation.

Students felt that the manikin improved their knowledge because they not only learnt about abnormal findings but also were provided with an opportunity to apply knowledge and interpret clinical signs, thus reiterating the knowledge required to examine a patient. Students also considered the feedback they received to be useful. Students felt they were more aware of the issues around examining a patient after examining the manikin. We found that the number of students who reported they were confident increased significantly for differentiating between normal and abnormal clinical signs after simulation whereas this decreased for examining a patient in a clinical setting. It may be that examining in a new setting without previous exposure to the modality, and having to recall steps, apply their knowledge and to be able to explore abnormalities, can be challenging to students.

SimMan® has drawbacks during examination. Students are not able to communicate as effectively as they would with a live subject. Also when using the manikin some aspects of the examination cannot be performed, for example, the head of the manikin cannot be turned when looking for JVP, neither can the wrist be bent to examine for flapping tremors, hence some students gave negative responses for using the manikin to practice examination. Some students specifically commented that it impacted negatively because they were examining the manikin for the first time. This may explain why students scored higher during peer examination when compared to examining the manikin. In the study by Swamy et al. in 2013 [13], it was found that graduate-entry students scored higher or equal to peer examination whilst performing chest examination on the manikin. This could be because they were mature students and thus the drawbacks felt by the students of examining the manikin were not as much of a hindrance. However, some students were less stressed because there was no shame or shyness or inhibition that may be associated with examining peers or patients. Students suggested that the session could be improved by increasing the time allocated, providing more teaching, and increasing the number of stations. They recommended frequent use of the manikin. Although SimMan® and other simulators like Harvey® and Ventriculoscope have their own advantages and disadvantages, SimMan® provides a more realistic and holistic experience of examining a patient [13].

Learners’ engagement with the repetitive practice of skills and assessment with specific informative feedback in a controlled setting has been shown to help in the acquisition of expertise [5]. Students evaluated the session incorporating both peer and SimMan® very positively. Together they can establish a strong foundation of clinical skills through experiential learning which can eventually be developed in clinical practice. Also it prepares students for future encounters with the manikin which is likely to increase in medical training, and helps them to acquire advanced clinical skills without having any negative impact on their learning experience at that stage.

Some limitations of this study include the fact that this is a carried out with a single cohort of medical students from a single school. SimMan®, a simulated patient cannot replace the learning experience that is derived from real patients in clinical practice. But, providing real patients may not be practical in preclinical years with respect to feasibility, accessibility and availability [13]. However, it encourages learning in an environment that does not compromise patient safety and can be useful to prepare students for clinical practice.

## Conclusion

The manikin provides students with an opportunity to experience examining patients during their early years of training. It reinforces the importance of eliciting normal findings and helps application of knowledge and skills in a safe environment. The findings of this study show that the manikin can be used as an adjunct in facilitating the acquisition of clinical skills during the early years of training as it improves students’ knowledge and confidence. The majority of the students found it useful to practice examination. It can be used to formatively assess student performance. Thus, an integrated learning approach using SimMan® as an adjunct to peer examination can benefit preclinical medical students’ ability to acquire clinical skills.
